# *catena*-Poly[[(tetra­hydro­furan-κ*O*)potas­sium]-di-μ-dimethyl­amido-κ^4^ *N*:*N*-aluminium-di-μ-dimethyl­amido-κ^4^ *N*:*N*-potassium-di-μ-dimethyl­amido-κ^4^ *N*:*N*-aluminium-di-μ-dimethyl­amido-κ^4^ *N*:*N*]

**DOI:** 10.1107/S1600536813003553

**Published:** 2013-02-13

**Authors:** Andrew Purdy, Damon Parrish

**Affiliations:** a Naval Research Laboratory, 4555 Overlook Av, SW, Washington, DC 20375, USA

## Abstract

The title compound, [Al_2_K_2_(C_2_H_6_N)_8_(C_4_H_8_O)]_*n*_, formed during the sonochemical reaction between Al(NMe_2_)_3_ and sodium–potassium alloy in the presence of tetra­hydro­furan (THF). Its asymmetric unit has two inequivalent K^+^ sites. One site is coordinated by a THF ligand, and crystallizes as a one-dimensional polymer with a backbone of catenated AlN_2_K rings. A twofold rotation axis bis­ects one K^+^ site and the THF ligand; the second K^+^ site is situated on an inversion centre, resulting in a planar four-coordination by N atoms. The latter symmetry operation generates the second half of the THF mol­ecule and fills out the coordination sphere of the potassium sites. The chains extend along the *c*-axis direction and zigzag at the THF-coordinated K^+^ sites by an angle of 106.02 (5)°.

## Related literature

Several (*RR*′N)_4_Al*M* (*M* = Li, Na) compounds that have catenated AlN_2_ *M* chains have been reported previously (Eisler & Chivers, 2006 ▶); Rings *et al.* 2000 ▶). The structure of tris­(dimethyl­amino)­aluminium is a mol­ecular dimer, see: Ouzounis *et al.* (1983 ▶). Heavily solvated tetra­amino­alanates tend to have separated cations instead of catenated chain structures, see: Hensen *et al.* (1999 ▶). For details of the synthesis, see: Ruff (1960 ▶); Ouzounis *et al.* (1983 ▶). 
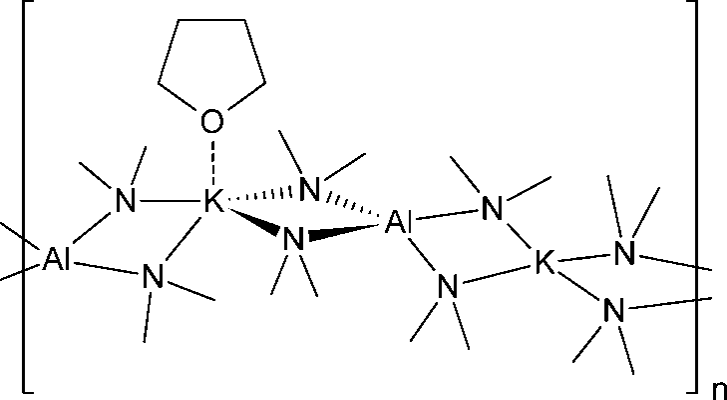


## Experimental

### 

#### Crystal data

[Al_2_K_2_(C_2_H_6_N)_8_(C_4_H_8_O)]
*M* *_r_* = 278.44
Monoclinic, 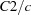

*a* = 13.247 (4) Å
*b* = 12.075 (4) Å
*c* = 20.822 (7) Å
β = 90.979 (5)°
*V* = 3330.1 (19) Å^3^
*Z* = 8
Mo *K*α radiation
μ = 0.36 mm^−1^
*T* = 173 K
0.74 × 0.57 × 0.42 mm

#### Data collection

Bruker SMART APEXII CCD diffractometer
Absorption correction: multi-scan (*SADABS*; Bruker, 2008 ▶) *T*_min_ = 0.776, *T*_max_ = 0.863
18795 measured reflections
4928 independent reflections
3986 reflections with *I* > 2σ(*I*)
*R*_int_ = 0.023

#### Refinement

*R*[*F* ^2^ > 2σ(*F* ^2^)] = 0.036
*wR*(*F* ^2^) = 0.107
*S* = 1.03
4928 reflections
155 parameters
H-atom parameters constrained
Δρ_max_ = 0.25 e Å^−3^
Δρ_min_ = −0.30 e Å^−3^

### 

Data collection: *APEX2* (Bruker, 2008 ▶); cell refinement: *SAINT* (Bruker, 2008 ▶); data reduction: *SAINT*; program(s) used to solve structure: *SHELXTL* (Sheldrick, 2008 ▶); program(s) used to refine structure: *SHELXTL*; molecular graphics: *SHELXTL*; software used to prepare material for publication: *SHELXTL*.

## Supplementary Material

Crystal structure: contains datablock(s) I, global. DOI: 10.1107/S1600536813003553/nk2195sup1.cif

Structure factors: contains datablock(s) I. DOI: 10.1107/S1600536813003553/nk2195Isup2.hkl

Additional supplementary materials:  crystallographic information; 3D view; checkCIF report

## Figures and Tables

**Fig. 1.**
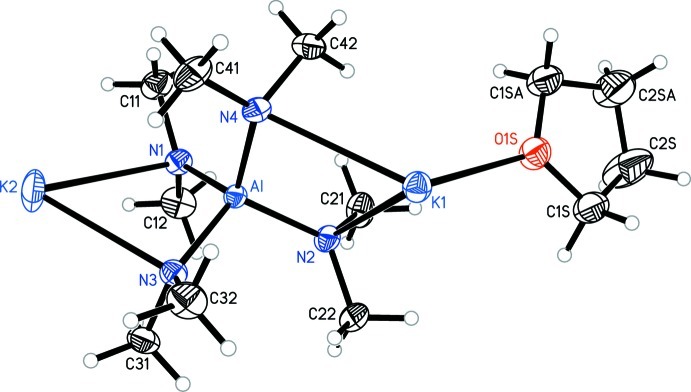
ORTEP diagram of the asymmetric unit, expanded to show the complete THF ligand. Symmetry code: A, -x+1, y, -z+3/2. Ellipsoids are at the 50& probability level.

**Fig. 2.**
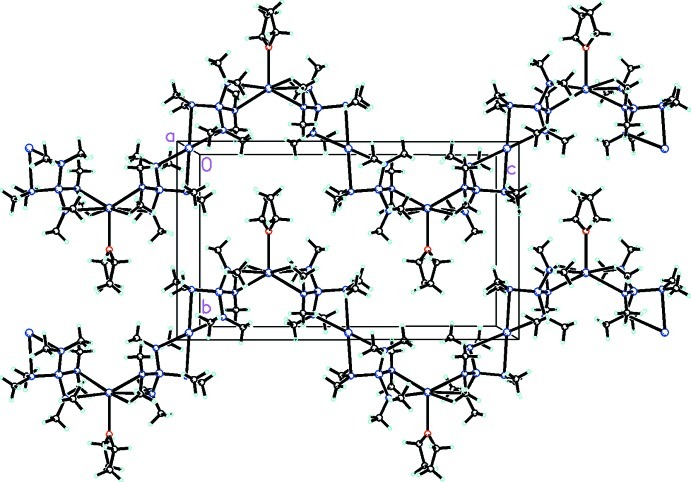
Packing diagram illustrating the 1-D polymeric chains.

## supplementary crystallographic information

### Comment

The title compound consists of an alternating chain of aluminium and potassium ions linked by bridging N atoms into a series of catenated 4-membered rings. One potassium site, K2, is 4-coordinate and planar, whereas a tetrahydrofuran (THF) ligand gives K1 a 5-coordinate twisted geometry. The chain extends along *c* and zigzags along *b* by an angle of 106.02° at each K1 atom. All N—Al—N angles are within 3° of the tetrahedral angle, and the Al—N distances of 1.838 (1) - 1.855 (1) Å are within the normal range for covalent Al—N bonds (Hensen, *et al.*, 1999). Similar one-dimensional polymeric chain structures have been observed in the Li and Na tetraamidoaluminates MAl(NH(CHMe_2_)_4_)_4_ (*M*= Li or Na), and Na(THF)Al(NHp-Tol)_4_ (Eisler and Chivers, 2006). An alkali amidoaluminate with less sterically demanding ligands, LiAl(NHMe)_4_ (Rings, *et al.* 2000), is coordinated in a three-dimensional polymeric framework. Amidoaluminates with fully solvated alkali cations tend to have isolated ions instead (Hensen, *et al.*, 1999).

### Experimental

All manipulations were performed in an a drybox under argon with dry solvents or inside a closed bulb. Tris(dimethylamino)aluminium (Ruff, 1960) was prepared by slowly adding LiNMe_2_ (6.07 g, 0.119 mmol) to a solution of AlCl_3_ (5.24 g, 0.039 mmol) in 50 ml Et_2_O. After the exotherm was over, the reaction was sonicated for 3 h in a closed bulb and then all solvents were removed under vacuum. The solids were sublimed under dynamic vacuum at 70–90 °C for 12 h, affording 5.51 g A l(NMe_2_)_3_ (88%) on the cold finger. The ^1^H NMR matched reported literature (Ouzounis, *et al.*, 1983).

In an attempt to prepare aluminium nanoparticles a mixture of 1 g A l(NMe_2_)_3_ and 0.55 g Na/K (50:50 by wt) in 25 ml of toluene was sonicated for 1 day. Al-27 NMR showed no evidence for aluminium metal. About 5 ml THF was added and the mixture was sonicated for several more days. After filtration in the drybox, the orange-brown solution was allowed to slowly evaporate in a vial. Crystals of the title compound slowly formed.

### Refinement

The full-matrix least-squares refinement on F2 included atomic coordinates and anisotropic thermal parameters for all non-H atoms. The H atoms were included using a riding model, with C—H distances of 0.98 angstroms for the methyl groups, and 0.99 angstroms for the THF molecule.

### Crystal data

**Table:**

[Al_2_K_2_(C_2_H_6_N)_8_(C_4_H_8_O)]	*F*(000) = 1216
*M**_r_* = 556.88	*D*_x_ = 1.111 Mg m^−^^3^
Monoclinic, *C*2/*c*	Mo *K*α radiation, λ = 0.71073 Å
*a* = 13.247 (4) Å	Cell parameters from 6485 reflections
*b* = 12.075 (4) Å	θ = 2.3–30.2°
*c* = 20.822 (7) Å	µ = 0.36 mm^−^^1^
β = 90.979 (5)°	*T* = 173 K
*V* = 3330.1 (19) Å^3^	Chunk, orange
*Z* = 4	0.74 × 0.57 × 0.42 mm

### Data collection

**Table:**

Bruker SMART APEXII CCD diffractometer	4928 independent reflections
Radiation source: fine-focus sealed tube	3986 reflections with *I* > 2σ(*I*)
Graphite monochromator	*R*_int_ = 0.023
ω scans	θ_max_ = 30.9°, θ_min_ = 2.0°
Absorption correction: multi-scan (*SADABS*; Bruker, 2008)	*h* = −18→18
*T*_min_ = 0.776, *T*_max_ = 0.863	*k* = −15→17
18795 measured reflections	*l* = −29→29

### Refinement

**Table:**

Refinement on *F*^2^	Primary atom site location: structure-invariant direct methods
Least-squares matrix: full	Secondary atom site location: difference Fourier map
*R*[*F*^2^ > 2σ(*F*^2^)] = 0.036	Hydrogen site location: inferred from neighbouring sites
*wR*(*F*^2^) = 0.107	H-atom parameters constrained
*S* = 1.03	*w* = 1/[σ^2^(*F*_o_^2^) + (0.0567*P*)^2^ + 0.9926*P*] where *P* = (*F*_o_^2^ + 2*F*_c_^2^)/3
4928 reflections	(Δ/σ)_max_ = 0.001
155 parameters	Δρ_max_ = 0.25 e Å^−^^3^
0 restraints	Δρ_min_ = −0.30 e Å^−^^3^

### Special details

**Table:**

Geometry. All e.s.d.'s (except the e.s.d. in the dihedral angle between two l.s. planes) are estimated using the full covariance matrix. The cell e.s.d.'s are taken into account individually in the estimation of e.s.d.'s in distances, angles and torsion angles; correlations between e.s.d.'s in cell parameters are only used when they are defined by crystal symmetry. An approximate (isotropic) treatment of cell e.s.d.'s is used for estimating e.s.d.'s involving l.s. planes.
Refinement. Refinement of *F*^2^ against ALL reflections. The weighted *R*-factor *wR* and goodness of fit *S* are based on *F*^2^, conventional *R*-factors *R* are based on *F*, with *F* set to zero for negative *F*^2^. The threshold expression of *F*^2^ > σ(*F*^2^) is used only for calculating *R*-factors(gt) *etc*. and is not relevant to the choice of reflections for refinement. *R*-factors based on *F*^2^ are statistically about twice as large as those based on *F*, and *R*- factors based on ALL data will be even larger.

### Fractional atomic coordinates and isotropic or equivalent isotropic displacement parameters (Å^2^)

**Table:**

	*x*	*y*	*z*	*U*_iso_*/*U*_eq_	
K1	0.5000	0.32471 (3)	0.7500	0.03918 (11)	
K2	0.5000	0.0000	0.5000	0.07103 (19)	
Al	0.52871 (2)	0.22259 (3)	0.595808 (16)	0.02748 (9)	
O1S	0.5000	0.54941 (12)	0.7500	0.0520 (3)	
N1	0.50523 (8)	0.23469 (9)	0.50846 (5)	0.0361 (2)	
N2	0.60258 (8)	0.34150 (9)	0.62545 (5)	0.0374 (2)	
N3	0.59582 (7)	0.09033 (8)	0.61187 (5)	0.0338 (2)	
N4	0.40721 (7)	0.21492 (9)	0.63840 (5)	0.0358 (2)	
C1S	0.57959 (13)	0.61739 (15)	0.77418 (9)	0.0614 (4)	
H1SA	0.6445	0.5961	0.7547	0.074*	
H1SB	0.5862	0.6096	0.8214	0.074*	
C2S	0.55312 (18)	0.73207 (19)	0.75688 (16)	0.1055 (10)	
H2SA	0.5692	0.7829	0.7929	0.127*	
H2SB	0.5910	0.7561	0.7188	0.127*	
C11	0.40625 (11)	0.24265 (14)	0.47861 (7)	0.0497 (3)	
H11A	0.4061	0.2036	0.4374	0.075*	
H11B	0.3893	0.3207	0.4715	0.075*	
H11C	0.3562	0.2090	0.5067	0.075*	
C12	0.58075 (12)	0.27839 (15)	0.46587 (7)	0.0543 (4)	
H12A	0.5837	0.2323	0.4272	0.081*	
H12B	0.6468	0.2781	0.4878	0.081*	
H12C	0.5629	0.3544	0.4537	0.081*	
C21	0.59360 (13)	0.44990 (12)	0.59569 (8)	0.0533 (4)	
H21A	0.6505	0.4617	0.5670	0.080*	
H21B	0.5942	0.5071	0.6291	0.080*	
H21C	0.5301	0.4540	0.5709	0.080*	
C22	0.69696 (10)	0.33235 (13)	0.66072 (8)	0.0501 (3)	
H22A	0.7531	0.3352	0.6307	0.075*	
H22B	0.6988	0.2619	0.6841	0.075*	
H22C	0.7030	0.3937	0.6913	0.075*	
C31	0.68988 (10)	0.06920 (12)	0.57922 (7)	0.0474 (3)	
H31A	0.6982	−0.0108	0.5731	0.057*	
H31B	0.7463	0.0979	0.6052	0.057*	
H31C	0.6885	0.1061	0.5373	0.057*	
C32	0.59875 (13)	0.03606 (14)	0.67380 (7)	0.0561 (4)	
H32A	0.6057	−0.0440	0.6677	0.084*	
H32B	0.5361	0.0516	0.6965	0.084*	
H32C	0.6564	0.0639	0.6991	0.084*	
C41	0.34632 (11)	0.11493 (15)	0.63898 (8)	0.0555 (4)	
H41A	0.3036	0.1123	0.6001	0.083*	
H41B	0.3036	0.1151	0.6769	0.083*	
H41C	0.3906	0.0500	0.6403	0.083*	
C42	0.34249 (10)	0.31184 (15)	0.63825 (8)	0.0526 (4)	
H42A	0.2979	0.3102	0.6002	0.079*	
H42B	0.3841	0.3789	0.6373	0.079*	
H42C	0.3017	0.3119	0.6771	0.079*	

### Atomic displacement parameters (Å^2^)

**Table:**

	*U*^11^	*U*^22^	*U*^33^	*U*^12^	*U*^13^	*U*^23^
K1	0.0424 (2)	0.0394 (2)	0.03566 (19)	0.000	−0.00112 (14)	0.000
K2	0.1055 (4)	0.0351 (2)	0.0709 (3)	0.0086 (3)	−0.0445 (3)	−0.0172 (2)
Al	0.02602 (16)	0.02410 (17)	0.03224 (17)	−0.00161 (12)	−0.00140 (12)	−0.00017 (12)
O1S	0.0558 (8)	0.0413 (8)	0.0586 (9)	0.000	−0.0084 (7)	0.000
N1	0.0381 (5)	0.0367 (6)	0.0335 (5)	0.0005 (4)	−0.0032 (4)	0.0051 (4)
N2	0.0341 (5)	0.0293 (5)	0.0488 (6)	−0.0068 (4)	−0.0032 (4)	−0.0014 (4)
N3	0.0348 (5)	0.0297 (5)	0.0369 (5)	0.0023 (4)	−0.0034 (4)	0.0035 (4)
N4	0.0268 (4)	0.0388 (6)	0.0418 (5)	−0.0039 (4)	0.0001 (4)	−0.0010 (4)
C1S	0.0516 (8)	0.0589 (10)	0.0732 (11)	−0.0001 (7)	−0.0148 (7)	−0.0021 (8)
C2S	0.0924 (17)	0.0547 (13)	0.167 (3)	−0.0215 (11)	−0.0594 (17)	0.0213 (14)
C11	0.0500 (8)	0.0513 (8)	0.0473 (7)	0.0007 (6)	−0.0152 (6)	0.0074 (6)
C12	0.0571 (9)	0.0629 (10)	0.0433 (7)	0.0029 (7)	0.0091 (6)	0.0151 (7)
C21	0.0656 (9)	0.0294 (7)	0.0650 (9)	−0.0119 (6)	0.0007 (7)	0.0004 (6)
C22	0.0353 (6)	0.0531 (9)	0.0615 (9)	−0.0094 (6)	−0.0054 (6)	−0.0121 (7)
C31	0.0379 (6)	0.0431 (8)	0.0611 (8)	0.0102 (6)	−0.0014 (6)	0.0014 (6)
C32	0.0657 (10)	0.0530 (9)	0.0494 (8)	0.0089 (7)	−0.0050 (7)	0.0171 (7)
C41	0.0431 (7)	0.0640 (10)	0.0593 (9)	−0.0239 (7)	0.0007 (6)	0.0040 (7)
C42	0.0341 (6)	0.0654 (10)	0.0581 (9)	0.0127 (6)	−0.0050 (6)	−0.0092 (7)

### Geometric parameters (Å, º)

**Table:**

K1—O1S	2.7133 (18)	N3—C31	1.4521 (17)
K1—N4^i^	2.9274 (12)	N4—C42	1.4506 (18)
K1—N4	2.9275 (12)	N4—C41	1.4522 (18)
K1—N2	2.9553 (14)	C1S—C2S	1.472 (3)
K1—N2^i^	2.9553 (14)	C1S—H1SA	0.9900
K1—C42^i^	3.1026 (16)	C1S—H1SB	0.9900
K1—C42	3.1026 (16)	C2S—C2S^i^	1.431 (4)
K1—C22^i^	3.2306 (17)	C2S—H2SA	0.9900
K1—C22	3.2307 (17)	C2S—H2SB	0.9900
K1—Al^i^	3.4662 (11)	C11—H11A	0.9800
K1—Al	3.4662 (11)	C11—H11B	0.9800
K2—N1	2.8402 (14)	C11—H11C	0.9800
K2—N1^ii^	2.8402 (14)	C12—H12A	0.9800
K2—N3^ii^	2.8506 (12)	C12—H12B	0.9800
K2—N3	2.8506 (12)	C12—H12C	0.9800
K2—C31^ii^	3.0990 (16)	C21—H21A	0.9800
K2—C31	3.0991 (16)	C21—H21B	0.9800
K2—C11^ii^	3.2103 (18)	C21—H21C	0.9800
K2—C11	3.2103 (18)	C22—H22A	0.9800
K2—Al^ii^	3.3649 (9)	C22—H22B	0.9800
K2—Al	3.3649 (9)	C22—H22C	0.9800
K2—H31A	3.0156	C31—H31A	0.9807
K2—H31C	2.9013	C31—H31B	0.9791
Al—N2	1.8383 (11)	C31—H31C	0.9800
Al—N1	1.8457 (12)	C32—H32A	0.9800
Al—N4	1.8533 (11)	C32—H32B	0.9800
Al—N3	1.8554 (11)	C32—H32C	0.9800
O1S—C1S	1.4215 (19)	C41—H41A	0.9800
O1S—C1S^i^	1.4215 (19)	C41—H41B	0.9800
N1—C11	1.4446 (17)	C41—H41C	0.9800
N1—C12	1.4478 (17)	C42—H42A	0.9800
N2—C22	1.4433 (17)	C42—H42B	0.9800
N2—C21	1.4522 (18)	C42—H42C	0.9800
N3—C32	1.4464 (17)		
			
O1S—K1—N4^i^	116.93 (2)	H31A—K2—H31C	31.3
O1S—K1—N4	116.93 (2)	N2—Al—N1	110.41 (5)
N4^i^—K1—N4	126.15 (5)	N2—Al—N4	109.88 (5)
O1S—K1—N2	86.06 (2)	N1—Al—N4	110.02 (5)
N4^i^—K1—N2	122.31 (3)	N2—Al—N3	111.17 (5)
N4—K1—N2	61.81 (3)	N1—Al—N3	108.54 (5)
O1S—K1—N2^i^	86.07 (2)	N4—Al—N3	106.74 (5)
N4^i^—K1—N2^i^	61.81 (3)	N2—Al—K2	151.29 (4)
N4—K1—N2^i^	122.31 (3)	N1—Al—K2	57.56 (4)
N2—K1—N2^i^	172.13 (4)	N4—Al—K2	98.83 (4)
O1S—K1—C42^i^	92.87 (3)	N3—Al—K2	57.88 (4)
N4^i^—K1—C42^i^	27.65 (4)	N2—Al—K1	58.49 (4)
N4—K1—C42^i^	147.19 (4)	N1—Al—K1	149.91 (4)
N2—K1—C42^i^	110.37 (4)	N4—Al—K1	57.63 (3)
N2^i^—K1—C42^i^	70.05 (4)	N3—Al—K1	101.48 (4)
O1S—K1—C42	92.87 (3)	K2—Al—K1	145.105 (13)
N4^i^—K1—C42	147.19 (4)	C1S—O1S—C1S^i^	109.46 (18)
N4—K1—C42	27.64 (4)	C1S—O1S—K1	125.27 (9)
N2—K1—C42	70.05 (4)	C1S^i^—O1S—K1	125.27 (9)
N2^i^—K1—C42	110.37 (4)	C11—N1—C12	110.11 (11)
C42^i^—K1—C42	174.26 (7)	C11—N1—Al	124.45 (9)
O1S—K1—C22^i^	88.36 (3)	C12—N1—Al	121.72 (9)
N4^i^—K1—C22^i^	83.60 (4)	C11—N1—K2	91.07 (8)
N4—K1—C22^i^	97.90 (4)	C12—N1—K2	110.00 (9)
N2—K1—C22^i^	152.99 (3)	Al—N1—K2	89.18 (4)
N2^i^—K1—C22^i^	26.51 (3)	C22—N2—C21	110.49 (11)
C42^i^—K1—C22^i^	96.27 (4)	C22—N2—Al	124.23 (9)
C42—K1—C22^i^	83.89 (5)	C21—N2—Al	121.43 (9)
O1S—K1—C22	88.36 (3)	C22—N2—K1	87.45 (8)
N4^i^—K1—C22	97.90 (4)	C21—N2—K1	113.62 (9)
N4—K1—C22	83.60 (4)	Al—N2—K1	89.48 (4)
N2—K1—C22	26.51 (3)	C32—N3—C31	109.13 (11)
N2^i^—K1—C22	153.00 (3)	C32—N3—Al	123.79 (10)
C42^i^—K1—C22	83.89 (5)	C31—N3—Al	118.63 (9)
C42—K1—C22	96.27 (5)	C32—N3—K2	124.05 (9)
C22^i^—K1—C22	176.73 (6)	C31—N3—K2	85.63 (8)
O1S—K1—Al^i^	110.839 (12)	Al—N3—K2	88.67 (4)
N4^i^—K1—Al^i^	32.32 (2)	C42—N4—C41	110.02 (12)
N4—K1—Al^i^	121.53 (3)	C42—N4—Al	118.47 (10)
N2—K1—Al^i^	153.72 (2)	C41—N4—Al	122.18 (10)
N2^i^—K1—Al^i^	32.03 (2)	C42—N4—K1	82.91 (8)
C42^i^—K1—Al^i^	50.99 (3)	C41—N4—K1	126.56 (9)
C42—K1—Al^i^	126.43 (4)	Al—N4—K1	90.05 (4)
C22^i^—K1—Al^i^	51.29 (3)	O1S—C1S—C2S	106.52 (14)
C22—K1—Al^i^	130.21 (3)	O1S—C1S—H1SA	110.4
O1S—K1—Al	110.838 (12)	C2S—C1S—H1SA	110.4
N4^i^—K1—Al	121.53 (3)	O1S—C1S—H1SB	110.4
N4—K1—Al	32.32 (2)	C2S—C1S—H1SB	110.4
N2—K1—Al	32.03 (2)	H1SA—C1S—H1SB	108.6
N2^i^—K1—Al	153.72 (3)	C2S^i^—C2S—C1S	106.16 (14)
C42^i^—K1—Al	126.43 (4)	C2S^i^—C2S—H2SA	110.5
C42—K1—Al	50.99 (3)	C1S—C2S—H2SA	110.5
C22^i^—K1—Al	130.21 (3)	C2S^i^—C2S—H2SB	110.5
C22—K1—Al	51.29 (3)	C1S—C2S—H2SB	110.5
Al^i^—K1—Al	138.32 (2)	H2SA—C2S—H2SB	108.7
N1—K2—N1^ii^	180.0	N1—C11—K2	62.19 (7)
N1—K2—N3^ii^	116.27 (3)	N1—C11—H11A	109.5
N1^ii^—K2—N3^ii^	63.73 (3)	K2—C11—H11A	71.2
N1—K2—N3	63.73 (3)	N1—C11—H11B	109.5
N1^ii^—K2—N3	116.26 (3)	K2—C11—H11B	170.5
N3^ii^—K2—N3	179.999 (1)	H11A—C11—H11B	109.5
N1—K2—C31^ii^	108.71 (3)	N1—C11—H11C	109.5
N1^ii^—K2—C31^ii^	71.29 (3)	K2—C11—H11C	78.6
N3^ii^—K2—C31^ii^	27.85 (3)	H11A—C11—H11C	109.5
N3—K2—C31^ii^	152.15 (3)	H11B—C11—H11C	109.5
N1—K2—C31	71.29 (3)	N1—C12—H12A	109.5
N1^ii^—K2—C31	108.71 (3)	N1—C12—H12B	109.5
N3^ii^—K2—C31	152.15 (3)	H12A—C12—H12B	109.5
N3—K2—C31	27.85 (3)	N1—C12—H12C	109.5
C31^ii^—K2—C31	180.0	H12A—C12—H12C	109.5
N1—K2—C11^ii^	153.26 (3)	H12B—C12—H12C	109.5
N1^ii^—K2—C11^ii^	26.74 (3)	N2—C21—H21A	109.5
N3^ii^—K2—C11^ii^	85.98 (4)	N2—C21—H21B	109.5
N3—K2—C11^ii^	94.02 (4)	H21A—C21—H21B	109.5
C31^ii^—K2—C11^ii^	97.83 (4)	N2—C21—H21C	109.5
C31—K2—C11^ii^	82.17 (4)	H21A—C21—H21C	109.5
N1—K2—C11	26.74 (3)	H21B—C21—H21C	109.5
N1^ii^—K2—C11	153.26 (3)	N2—C22—K1	66.04 (7)
N3^ii^—K2—C11	94.02 (4)	N2—C22—H22A	109.5
N3—K2—C11	85.98 (4)	K1—C22—H22A	175.5
C31^ii^—K2—C11	82.17 (4)	N2—C22—H22B	109.5
C31—K2—C11	97.83 (4)	K1—C22—H22B	72.8
C11^ii^—K2—C11	180.0	H22A—C22—H22B	109.5
N1—K2—Al^ii^	146.74 (2)	N2—C22—H22C	109.5
N1^ii^—K2—Al^ii^	33.26 (2)	K1—C22—H22C	72.9
N3^ii^—K2—Al^ii^	33.45 (2)	H22A—C22—H22C	109.5
N3—K2—Al^ii^	146.55 (2)	H22B—C22—H22C	109.5
C31^ii^—K2—Al^ii^	52.16 (3)	N3—C31—K2	66.52 (7)
C31—K2—Al^ii^	127.84 (3)	N3—C31—H31A	109.5
C11^ii^—K2—Al^ii^	52.61 (3)	K2—C31—H31A	76.0
C11—K2—Al^ii^	127.39 (3)	N3—C31—H31B	109.4
N1—K2—Al	33.26 (2)	K2—C31—H31B	174.3
N1^ii^—K2—Al	146.74 (2)	H31A—C31—H31B	109.5
N3^ii^—K2—Al	146.55 (2)	N3—C31—H31C	109.5
N3—K2—Al	33.45 (2)	K2—C31—H31C	69.3
C31^ii^—K2—Al	127.84 (3)	H31A—C31—H31C	109.4
C31—K2—Al	52.16 (3)	H31B—C31—H31C	109.4
C11^ii^—K2—Al	127.39 (3)	N3—C32—H32A	109.5
C11—K2—Al	52.61 (3)	N3—C32—H32B	109.5
Al^ii^—K2—Al	180.0	H32A—C32—H32B	109.5
N1—K2—H31A	89.5	N3—C32—H32C	109.5
N1^ii^—K2—H31A	90.5	H32A—C32—H32C	109.5
N3^ii^—K2—H31A	140.2	H32B—C32—H32C	109.5
N3—K2—H31A	39.8	N4—C41—H41A	109.5
C31^ii^—K2—H31A	161.6	N4—C41—H41B	109.5
C31—K2—H31A	18.4	H41A—C41—H41B	109.5
C11^ii^—K2—H31A	63.8	N4—C41—H41C	109.5
C11—K2—H31A	116.2	H41A—C41—H41C	109.5
Al^ii^—K2—H31A	110.7	H41B—C41—H41C	109.5
Al—K2—H31A	69.3	N4—C42—K1	69.45 (7)
N1—K2—H31C	61.5	N4—C42—H42A	109.5
N1^ii^—K2—H31C	118.5	K1—C42—H42A	174.5
N3^ii^—K2—H31C	139.2	N4—C42—H42B	109.5
N3—K2—H31C	40.8	K1—C42—H42B	66.5
C31^ii^—K2—H31C	161.6	H42A—C42—H42B	109.5
C31—K2—H31C	18.4	N4—C42—H42C	109.5
C11^ii^—K2—H31C	92.1	K1—C42—H42C	75.8
C11—K2—H31C	87.9	H42A—C42—H42C	109.5
Al^ii^—K2—H31C	126.8	H42B—C42—H42C	109.5
Al—K2—H31C	53.2		
			
N1—K2—Al—N2	−72.54 (9)	K1—Al—N2—C21	−117.65 (12)
N1^ii^—K2—Al—N2	107.46 (9)	N1—Al—N2—K1	148.73 (4)
N3^ii^—K2—Al—N2	−104.99 (9)	N4—Al—N2—K1	27.20 (5)
N3—K2—Al—N2	75.01 (9)	N3—Al—N2—K1	−90.74 (5)
C31^ii^—K2—Al—N2	−136.20 (9)	K2—Al—N2—K1	−152.06 (5)
C31—K2—Al—N2	43.80 (9)	O1S—K1—N2—C22	94.23 (7)
C11^ii^—K2—Al—N2	79.62 (9)	N4^i^—K1—N2—C22	−25.34 (9)
C11—K2—Al—N2	−100.37 (9)	N4—K1—N2—C22	−142.28 (9)
Al^ii^—K2—Al—N2	−90.4 (6)	N2^i^—K1—N2—C22	94.24 (8)
N1^ii^—K2—Al—N1	180.0	C42^i^—K1—N2—C22	2.63 (9)
N3^ii^—K2—Al—N1	−32.45 (6)	C42—K1—N2—C22	−171.27 (9)
N3—K2—Al—N1	147.55 (6)	C22^i^—K1—N2—C22	172.81 (13)
C31^ii^—K2—Al—N1	−63.66 (5)	Al^i^—K1—N2—C22	−37.53 (10)
C31—K2—Al—N1	116.34 (5)	Al—K1—N2—C22	−124.29 (9)
C11^ii^—K2—Al—N1	152.16 (5)	O1S—K1—N2—C21	−17.06 (9)
C11—K2—Al—N1	−27.84 (5)	N4^i^—K1—N2—C21	−136.63 (9)
Al^ii^—K2—Al—N1	−17.8 (6)	N4—K1—N2—C21	106.44 (9)
N1—K2—Al—N4	108.17 (5)	N2^i^—K1—N2—C21	−17.05 (9)
N1^ii^—K2—Al—N4	−71.83 (5)	C42^i^—K1—N2—C21	−108.66 (9)
N3^ii^—K2—Al—N4	75.72 (5)	C42—K1—N2—C21	77.44 (9)
N3—K2—Al—N4	−104.28 (5)	C22^i^—K1—N2—C21	61.52 (13)
C31^ii^—K2—Al—N4	44.51 (5)	C22—K1—N2—C21	−111.29 (12)
C31—K2—Al—N4	−135.49 (5)	Al^i^—K1—N2—C21	−148.81 (8)
C11^ii^—K2—Al—N4	−99.67 (5)	Al—K1—N2—C21	124.42 (10)
C11—K2—Al—N4	80.33 (5)	O1S—K1—N2—Al	−141.47 (4)
Al^ii^—K2—Al—N4	90.3 (6)	N4^i^—K1—N2—Al	98.95 (5)
N1—K2—Al—N3	−147.55 (6)	N4—K1—N2—Al	−17.98 (3)
N1^ii^—K2—Al—N3	32.45 (6)	N2^i^—K1—N2—Al	−141.47 (4)
N3^ii^—K2—Al—N3	179.998 (1)	C42^i^—K1—N2—Al	126.92 (5)
C31^ii^—K2—Al—N3	148.79 (5)	C42—K1—N2—Al	−46.98 (5)
C31—K2—Al—N3	−31.21 (5)	C22^i^—K1—N2—Al	−62.89 (10)
C11^ii^—K2—Al—N3	4.61 (5)	C22—K1—N2—Al	124.29 (9)
C11—K2—Al—N3	−175.39 (5)	Al^i^—K1—N2—Al	86.77 (6)
Al^ii^—K2—Al—N3	−165.4 (5)	N2—Al—N3—C32	78.67 (12)
N1—K2—Al—K1	151.74 (5)	N1—Al—N3—C32	−159.71 (11)
N1^ii^—K2—Al—K1	−28.26 (5)	N4—Al—N3—C32	−41.15 (12)
N3^ii^—K2—Al—K1	119.29 (4)	K2—Al—N3—C32	−131.18 (12)
N3—K2—Al—K1	−60.71 (4)	K1—Al—N3—C32	18.22 (11)
C31^ii^—K2—Al—K1	88.08 (4)	N2—Al—N3—C31	−65.87 (11)
C31—K2—Al—K1	−91.92 (4)	N1—Al—N3—C31	55.76 (10)
C11^ii^—K2—Al—K1	−56.10 (4)	N4—Al—N3—C31	174.31 (9)
C11—K2—Al—K1	123.91 (4)	K2—Al—N3—C31	84.29 (10)
Al^ii^—K2—Al—K1	133.9 (5)	K1—Al—N3—C31	−126.31 (9)
O1S—K1—Al—N2	41.68 (4)	N2—Al—N3—K2	−150.16 (4)
N4^i^—K1—Al—N2	−101.64 (5)	N1—Al—N3—K2	−28.53 (5)
N4—K1—Al—N2	149.41 (6)	N4—Al—N3—K2	90.02 (5)
N2^i^—K1—Al—N2	168.89 (6)	K1—Al—N3—K2	149.395 (19)
C42^i^—K1—Al—N2	−68.66 (6)	N1—K2—N3—C32	150.13 (11)
C42—K1—Al—N2	117.83 (6)	N1^ii^—K2—N3—C32	−29.87 (11)
C22^i^—K1—Al—N2	148.04 (6)	N3^ii^—K2—N3—C32	10 (12)
C22—K1—Al—N2	−28.19 (5)	C31^ii^—K2—N3—C32	69.82 (13)
Al^i^—K1—Al—N2	−138.33 (4)	C31—K2—N3—C32	−110.18 (13)
O1S—K1—Al—N1	−34.33 (7)	C11^ii^—K2—N3—C32	−45.36 (11)
N4^i^—K1—Al—N1	−177.64 (7)	C11—K2—N3—C32	134.65 (11)
N4—K1—Al—N1	73.41 (8)	Al^ii^—K2—N3—C32	−49.03 (12)
N2—K1—Al—N1	−76.00 (8)	Al—K2—N3—C32	130.97 (12)
N2^i^—K1—Al—N1	92.89 (9)	N1—K2—N3—C31	−99.69 (8)
C42^i^—K1—Al—N1	−144.66 (8)	N1^ii^—K2—N3—C31	80.31 (8)
C42—K1—Al—N1	41.82 (8)	N3^ii^—K2—N3—C31	121 (12)
C22^i^—K1—Al—N1	72.04 (8)	C31^ii^—K2—N3—C31	180.0
C22—K1—Al—N1	−104.20 (8)	C11^ii^—K2—N3—C31	64.83 (8)
Al^i^—K1—Al—N1	145.67 (7)	C11—K2—N3—C31	−115.17 (8)
O1S—K1—Al—N4	−107.73 (4)	Al^ii^—K2—N3—C31	61.15 (8)
N4^i^—K1—Al—N4	108.95 (6)	Al—K2—N3—C31	−118.84 (8)
N2—K1—Al—N4	−149.41 (6)	N1—K2—N3—Al	19.16 (3)
N2^i^—K1—Al—N4	19.48 (7)	N1^ii^—K2—N3—Al	−160.84 (3)
C42^i^—K1—Al—N4	141.93 (6)	N3^ii^—K2—N3—Al	−121 (12)
C42—K1—Al—N4	−31.58 (6)	C31^ii^—K2—N3—Al	−61.15 (8)
C22^i^—K1—Al—N4	−1.37 (6)	C31—K2—N3—Al	118.84 (8)
C22—K1—Al—N4	−177.60 (6)	C11^ii^—K2—N3—Al	−176.33 (4)
Al^i^—K1—Al—N4	72.26 (4)	C11—K2—N3—Al	3.67 (4)
O1S—K1—Al—N3	149.60 (3)	Al^ii^—K2—N3—Al	180.0
N4^i^—K1—Al—N3	6.28 (4)	N2—Al—N4—C42	54.48 (11)
N4—K1—Al—N3	−102.67 (5)	N1—Al—N4—C42	−67.29 (11)
N2—K1—Al—N3	107.92 (6)	N3—Al—N4—C42	175.13 (9)
N2^i^—K1—Al—N3	−83.18 (6)	K2—Al—N4—C42	−125.88 (9)
C42^i^—K1—Al—N3	39.26 (5)	K1—Al—N4—C42	81.96 (10)
C42—K1—Al—N3	−134.25 (6)	N2—Al—N4—C41	−162.25 (10)
C22^i^—K1—Al—N3	−104.04 (5)	N1—Al—N4—C41	75.98 (12)
C22—K1—Al—N3	79.73 (5)	N3—Al—N4—C41	−41.60 (12)
Al^i^—K1—Al—N3	−30.40 (3)	K2—Al—N4—C41	17.39 (11)
O1S—K1—Al—K2	−161.489 (17)	K1—Al—N4—C41	−134.78 (12)
N4^i^—K1—Al—K2	55.20 (4)	N2—Al—N4—K1	−27.47 (5)
N4—K1—Al—K2	−53.75 (4)	N1—Al—N4—K1	−149.24 (4)
N2—K1—Al—K2	156.84 (5)	N3—Al—N4—K1	93.17 (5)
N2^i^—K1—Al—K2	−34.27 (6)	K2—Al—N4—K1	152.17 (2)
C42^i^—K1—Al—K2	88.18 (4)	O1S—K1—N4—C42	−31.94 (8)
C42—K1—Al—K2	−85.34 (5)	N4^i^—K1—N4—C42	148.06 (8)
C22^i^—K1—Al—K2	−55.12 (4)	N2—K1—N4—C42	−100.87 (9)
C22—K1—Al—K2	128.64 (4)	N2^i^—K1—N4—C42	71.36 (9)
Al^i^—K1—Al—K2	18.510 (17)	C42^i^—K1—N4—C42	175.00 (6)
N4^i^—K1—O1S—C1S	37.19 (10)	C22^i^—K1—N4—C42	60.24 (8)
N4—K1—O1S—C1S	−142.81 (10)	C22—K1—N4—C42	−116.82 (9)
N2—K1—O1S—C1S	−87.28 (10)	Al^i^—K1—N4—C42	109.28 (8)
N2^i^—K1—O1S—C1S	92.72 (10)	Al—K1—N4—C42	−118.70 (10)
C42^i^—K1—O1S—C1S	22.96 (10)	O1S—K1—N4—C41	−141.66 (11)
C42—K1—O1S—C1S	−157.04 (10)	N4^i^—K1—N4—C41	38.34 (11)
C22^i^—K1—O1S—C1S	119.16 (10)	N2—K1—N4—C41	149.41 (12)
C22—K1—O1S—C1S	−60.84 (10)	N2^i^—K1—N4—C41	−38.35 (12)
Al^i^—K1—O1S—C1S	72.02 (10)	C42^i^—K1—N4—C41	65.28 (13)
Al—K1—O1S—C1S	−107.98 (10)	C42—K1—N4—C41	−109.71 (14)
N4^i^—K1—O1S—C1S^i^	−142.81 (10)	C22^i^—K1—N4—C41	−49.47 (12)
N4—K1—O1S—C1S^i^	37.19 (10)	C22—K1—N4—C41	133.46 (12)
N2—K1—O1S—C1S^i^	92.72 (10)	Al^i^—K1—N4—C41	−0.43 (12)
N2^i^—K1—O1S—C1S^i^	−87.28 (10)	Al—K1—N4—C41	131.58 (13)
C42^i^—K1—O1S—C1S^i^	−157.04 (10)	O1S—K1—N4—Al	86.76 (4)
C42—K1—O1S—C1S^i^	22.96 (10)	N4^i^—K1—N4—Al	−93.24 (4)
C22^i^—K1—O1S—C1S^i^	−60.84 (10)	N2—K1—N4—Al	17.83 (3)
C22—K1—O1S—C1S^i^	119.16 (10)	N2^i^—K1—N4—Al	−169.94 (4)
Al^i^—K1—O1S—C1S^i^	−107.98 (10)	C42^i^—K1—N4—Al	−66.30 (8)
Al—K1—O1S—C1S^i^	72.02 (10)	C42—K1—N4—Al	118.70 (9)
N2—Al—N1—C11	−118.54 (11)	C22^i^—K1—N4—Al	178.95 (4)
N4—Al—N1—C11	2.91 (13)	C22—K1—N4—Al	1.88 (5)
N3—Al—N1—C11	119.37 (11)	Al^i^—K1—N4—Al	−132.01 (3)
K2—Al—N1—C11	90.73 (11)	C1S^i^—O1S—C1S—C2S	−7.13 (16)
K1—Al—N1—C11	−56.57 (15)	K1—O1S—C1S—C2S	172.87 (16)
N2—Al—N1—C12	37.57 (12)	O1S—C1S—C2S—C2S^i^	19.0 (4)
N4—Al—N1—C12	159.02 (11)	C12—N1—C11—K2	111.78 (11)
N3—Al—N1—C12	−84.52 (12)	Al—N1—C11—K2	−89.74 (9)
K2—Al—N1—C12	−113.16 (12)	N1^ii^—K2—C11—N1	180.0
K1—Al—N1—C12	99.54 (12)	N3^ii^—K2—C11—N1	−147.85 (8)
N2—Al—N1—K2	150.73 (4)	N3—K2—C11—N1	32.15 (8)
N4—Al—N1—K2	−87.82 (5)	C31^ii^—K2—C11—N1	−173.11 (8)
N3—Al—N1—K2	28.64 (5)	C31—K2—C11—N1	6.88 (8)
K1—Al—N1—K2	−147.30 (5)	C11^ii^—K2—C11—N1	−88 (10)
N1^ii^—K2—N1—C11	−116 (15)	Al^ii^—K2—C11—N1	−145.30 (6)
N3^ii^—K2—N1—C11	36.29 (8)	Al—K2—C11—N1	34.70 (6)
N3—K2—N1—C11	−143.71 (8)	C21—N2—C22—K1	114.31 (10)
C31^ii^—K2—N1—C11	7.20 (9)	Al—N2—C22—K1	−87.64 (9)
C31—K2—N1—C11	−172.80 (9)	O1S—K1—C22—N2	−84.46 (7)
C11^ii^—K2—N1—C11	180.0	N4^i^—K1—C22—N2	158.58 (7)
Al^ii^—K2—N1—C11	55.55 (9)	N4—K1—C22—N2	32.87 (7)
Al—K2—N1—C11	−124.45 (9)	N2^i^—K1—C22—N2	−162.50 (10)
N1^ii^—K2—N1—C12	132 (15)	C42^i^—K1—C22—N2	−177.52 (8)
N3^ii^—K2—N1—C12	−75.59 (9)	C42—K1—C22—N2	8.25 (8)
N3—K2—N1—C12	104.41 (9)	C22^i^—K1—C22—N2	−84.45 (7)
C31^ii^—K2—N1—C12	−104.68 (9)	Al^i^—K1—C22—N2	159.32 (6)
C31—K2—N1—C12	75.32 (9)	Al—K1—C22—N2	34.16 (6)
C11^ii^—K2—N1—C12	68.11 (12)	C32—N3—C31—K2	124.60 (11)
C11—K2—N1—C12	−111.88 (12)	Al—N3—C31—K2	−86.09 (7)
Al^ii^—K2—N1—C12	−56.33 (10)	N1—K2—C31—N3	68.95 (7)
Al—K2—N1—C12	123.67 (10)	N1^ii^—K2—C31—N3	−111.05 (7)
N1^ii^—K2—N1—Al	8 (15)	N3^ii^—K2—C31—N3	180.0
N3^ii^—K2—N1—Al	160.74 (3)	C31^ii^—K2—C31—N3	167 (13)
N3—K2—N1—Al	−19.26 (3)	C11^ii^—K2—C31—N3	−114.31 (8)
C31^ii^—K2—N1—Al	131.65 (4)	C11—K2—C31—N3	65.69 (8)
C31—K2—N1—Al	−48.35 (4)	Al^ii^—K2—C31—N3	−142.31 (6)
C11^ii^—K2—N1—Al	−55.56 (9)	Al—K2—C31—N3	37.69 (6)
C11—K2—N1—Al	124.45 (9)	C41—N4—C42—K1	126.41 (10)
Al^ii^—K2—N1—Al	180.0	Al—N4—C42—K1	−86.19 (7)
N1—Al—N2—C22	−124.71 (11)	O1S—K1—C42—N4	151.82 (7)
N4—Al—N2—C22	113.76 (11)	N4^i^—K1—C42—N4	−52.04 (12)
N3—Al—N2—C22	−4.18 (12)	N2—K1—C42—N4	67.05 (7)
K2—Al—N2—C22	−65.50 (15)	N2^i^—K1—C42—N4	−121.32 (7)
K1—Al—N2—C22	86.56 (11)	C42^i^—K1—C42—N4	−28.18 (8)
N1—Al—N2—C21	31.07 (12)	C22^i^—K1—C42—N4	−120.14 (8)
N4—Al—N2—C21	−90.46 (12)	C22—K1—C42—N4	63.15 (8)
N3—Al—N2—C21	151.60 (10)	Al^i^—K1—C42—N4	−89.52 (8)
K2—Al—N2—C21	90.28 (13)	Al—K1—C42—N4	37.12 (6)

Symmetry codes: (i) −*x*+1, *y*, −*z*+3/2; (ii) −*x*+1, −*y*, −*z*+1.
